# Magnetic Hyperthermia Enhancement in Iron‐based Materials Driven by Carbon Support Interactions

**DOI:** 10.1002/chem.202201861

**Published:** 2022-10-06

**Authors:** Lucía Vizcaíno‐Anaya, Carlos Herreros‐Lucas, José M. Vila‐Fungueiriño, María del Carmen Giménez‐López

**Affiliations:** ^1^ Centro Singular de Investigación en Química Biolóxica e Materiais Moleculares (CiQUS) Universidade de Santiago de Compostela 15782 Santiago de Compostela Spain

**Keywords:** carbon supports, iron magnetic nanoparticles, magnetic hyperthermia, nanoparticle-carbon interactions, specific absorption rates

## Abstract

Magnetic hyperthermia (MH) shows great potential in clinical applications because of its very localized action and minimal side effects. Because of their high saturation magnetization values, reduced forms of iron are promising candidates for MH. However, they must be protected in order to overcome their toxicity and instability (i. e., oxidation) under biological conditions. In this work, a novel methodology for the protection of iron nanoparticles through confinement within graphitic carbon layers after thermal treatment of preformed nanoparticles supported on carbon is reported. We demonstrate that the size and composition of the nascent confined iron nanoparticles, as well as the thickness of their protective carbon layer can be controlled by selecting the nature of the carbon support. Our findings reveal that a higher nanoparticle–carbon interaction, mediated by the presence of oxygen‐containing groups, induces the formation of small and well‐protected α‐Fe‐based nanoparticles that exhibit promising results towards MH based on their enhanced specific absorption rate values.

## Graphical abstract

**The control of both** the thickness of the protective carbon layer, and the size and composition of the nascent confined iron nanoparticles is driven by carbon support interactions. The iron hybrid materials (Fe_3_C@C/CNF_AC_) obtained from acid‐treated carbon supports exhibit enhanced specific absorption rate values in biocompatible media.

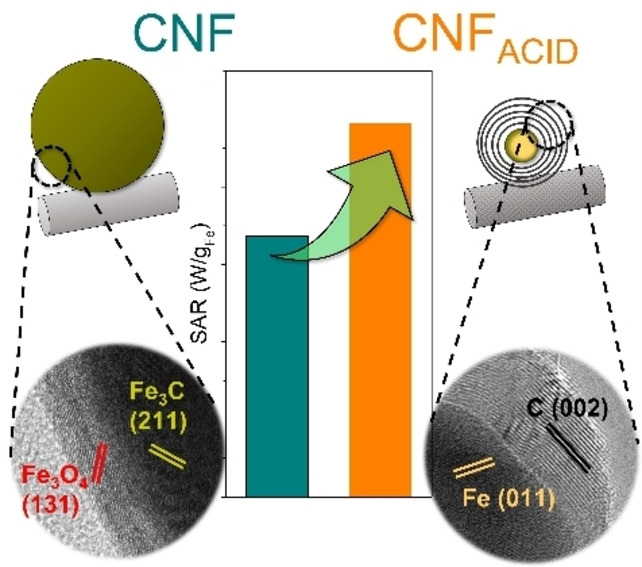

## Introduction

Nanomaterials are widely used in medicine because of their small size (1–100 nm), tunable properties, and ability to conjugate with therapeutic and diagnostic molecules.^[1]^ There are numerous research fields in biomedicine where nanomaterials carry out functions that traditional medicine cannot achieve, such as immunotherapy,[^2^, ^3^] phototherapy,^[4]^ chemo and photothermal therapies,[^5^, ^6^] antibacterial agents,^[7]^ imaging agents,^[8]^ disease treatment^[9]^, drug delivery,[^10^, ^11^, ^12^] and so on. Innovative developments in materials are incessantly needed to meet the increasing demands of healthcare.

Because of its localized action and minimal side effects, magnetic hyperthermia (MH) is a potential tool for cancer treatment. It consists of an induced local heating by applying an external alternating magnetic field to magnetic nanomaterials delivered into a tumour tissue, leaving in turn the surrounding tissue intact.[^13^, ^14^] A key parameter in MH is the amount of energy that is transformed into heat per mass of magnetic material, known as specific absorption rate (SAR).

Due to their biocompatibility and saturation magnetization, as well as their abundance, iron oxide‐based nanoparticles (Fe_3_O_4_ and Fe_2_O_3_) are the most widely used magnetic nanomaterials in MH.^[15]^ The magnetic performance of these nanomaterials can be directly influenced by several factors such as size, shape, doping, concentration, coating thickness, functionalization, among others.^[13]^ For instance, it has been demonstrated the existence of an optimal nanoparticle size to maximize SAR values. Ma et al.^[16]^ studied Fe_3_O_4_ particles of a wide range of sizes (from 7.5 to 416 nm) and found the highest magnetic coercivity and SAR values for samples of a mean diameter of 46 nm. On the other hand, an increase in SAR values can be achieved by cubic nanoparticles instead of the classical spherical ones due to the beneficial role of surface anisotropy,^[17]^ or by changing the composition through carbothermal treatments that create oxygen vacancies in iron oxide nanoparticles.^[18]^ In this context, reduced forms of iron nanoparticles, such as Fe and Fe_3_C, are very promising candidates for hyperthermia applications due to their higher saturation magnetization.^[19]^ However, these non‐oxide iron phases are easily oxidized when exposed to the ambient or under biological conditions, and they can become toxic^[20]^ so they must become strongly stabilized for medical applications.

A promising strategy for protecting and isolating from the ambient these reduced iron nanoparticles is to encapsulate them within different materials, including precious metal coatings, surfactants, polymers, silica or carbon, among others, being the later especially interesting due to its superior thermal and chemical stability, as well as biocompatibility.^[21]^ Among different carbon materials, carbon nanotubes have shown additional advantages as they possess an internal cavity that can host a wide variety of molecular nanostructures^[22]^ and their protection capacity against oxidation has been previously demonstrated.^[23]^ Various systems composed of Fe encapsulated within carbon nanotubes have been studied for hyperthermia applications.[^20^, ^24^, ^25^, ^26^] However, control of the parameters related to the hyperthermia performance of these systems (i. e., size, shape, particle distribution or carbon nanotube filling ratio) is still a major challenge to implement them in biomedical applications due to the complexity of the systems at the nanoscale.

Here, we report a novel methodology for controlling the protection of reduced forms of iron nanoparticles for their application in MH by confinement within graphitic carbon layers (Fe_3_C@C/CNF) upon thermal treatment of preformed Fe_3_O_4_ nanoparticles supported on modified carbon nanofibers (CNF). By selecting the nature of the CNF support, both the protective carbon thickness, and the size and composition of the nascent confined iron nanoparticles can be controlled. We show that CNF supports with a high number of defects limit the growth of iron nanoparticles during thermal treatment, inducing the formation of small and well‐protected α‐Fe‐based nanoparticles. The obtained iron‐based hybrid nanostructures show enhanced SAR values demonstrating a great potential for alternating current (AC) magnetic hyperthermia applications.

## Results and Discussion

**Scheme 1:**
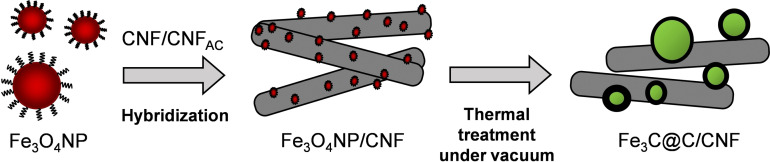
Schematic representation of the synthetic methodology to obtain Fe_3_C@C/CNF from thermal treatment of Fe_3_O_4_NP/CNF under vacuum.

The two‐step procedure synthesis of Fe_3_C@C/CNF is shown in Scheme 1. Briefly, ∼4 nm oleic acid‐stabilized iron oxide nanoparticles (Fe_3_O_4_NP, Figures S1–S4 in the Supporting Information) were first prepared using iron(III) acetylacetonate (Fe(acac)_3_) as precursor following a procedure reported by Sun et al.^[27]^ Then, these Fe_3_O_4_NP were supported on CNF and heated at high temperature (1000 °C) in vacuum to promote the simultaneous reduction of iron oxide to iron, and the consequently dissolution of carbon in iron. To investigate whether the structure and composition of the thermally annealed iron nanoparticles can be controlled by the interaction with the carbon support, two different types of carbon nanofibers (before (CNF) and after acid treatment (CNF_AC_), Figures S5–S8) were employed, enabling different nanoparticle–carbon interactions through functional groups and defects in the CNF support.

**Figure 1:**
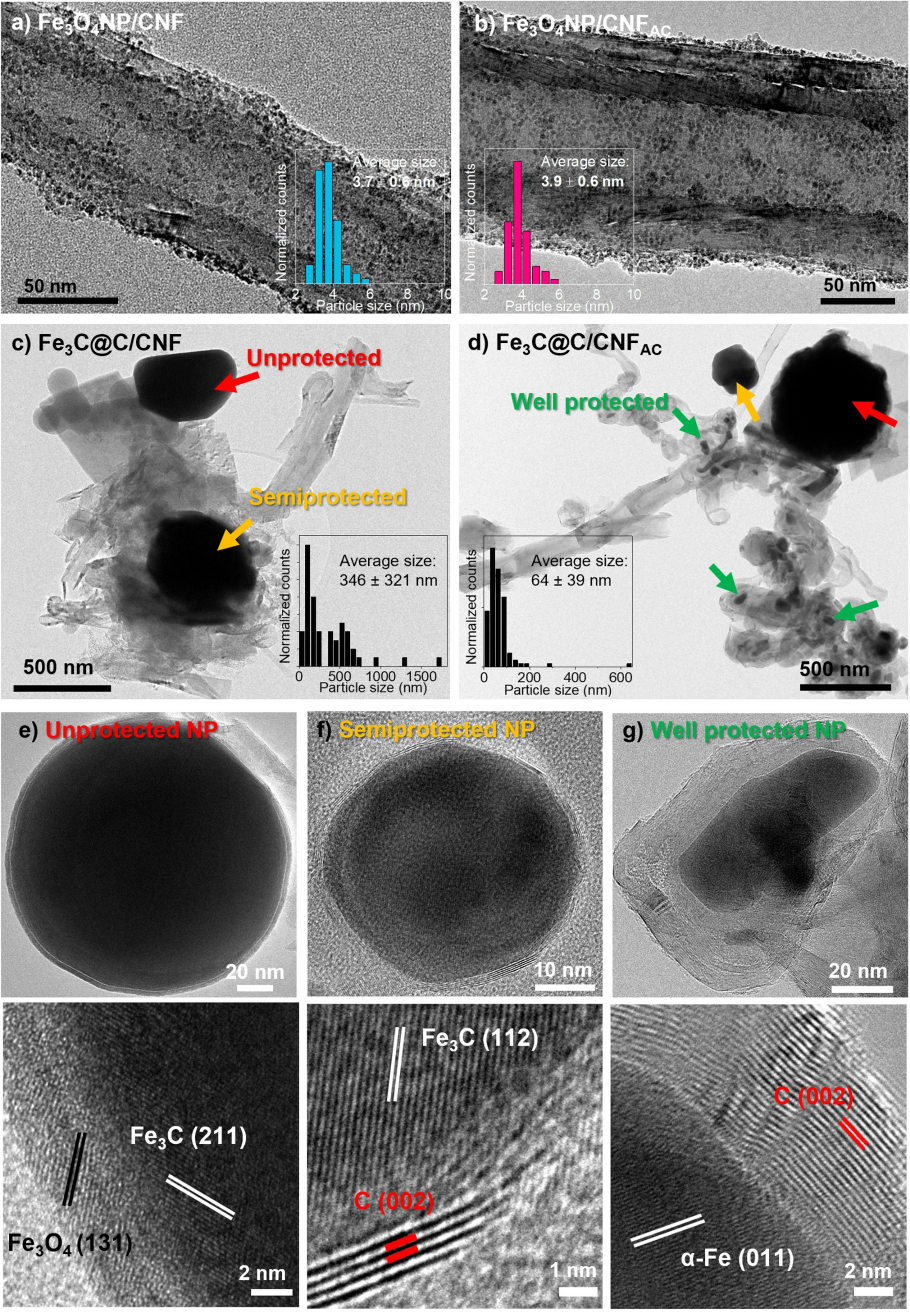
HRTEM images of a) Fe_3_O_4_NP/CNF and b) Fe_3_O_4_NP/CNF_AC_, and size distribution of Fe_3_O_4_NPs. HRTEM images of c) Fe_3_C@C/CNF and d) Fe_3_C@C/CNF_AC_ and size distribution of Fe_3_C@C. e)–g) HRTEM images of the three characteristic types of nanoparticles found in Fe_3_C@C/CNF_AC_ hybrid material with high magnification images showing interplanar distances for the different iron crystallographic phases.

Transmission electron microscopy (TEM) images of Fe_3_O_4_NP dispersed on CNF before thermal treatment (Figure 1a, b) reveal a similar distribution of Fe_3_O_4_ nanoparticles on the carbon surface of both types of carbon nanofibers (i. e., CNF and CNF_AC_) with no noticeable change on the nanoparticle size with respect to the free‐standing preformed nanoparticles (Figure S1b). It is observed that the synthetic protocol using iron(III) acetylacetonate as precursor is suitable for preparing pseudo‐spherical iron oxide nanoparticles with a very narrow size distribution (i. e., <1 nm, Figure 1a, b).

In contrast, TEM images of Fe_3_O_4_NP/CNF after thermal treatment (i. e., Fe_3_C@C/CNF) show the formation of larger nanoparticles. SEM images of annealed hybrids (Figures S9) further confirm the dramatic increase of the nanoparticle size with respect to the hybrid before thermal treatment (Figure S10). Furthermore, there is a big difference in chemical composition, as well as in the nanoparticle size between Fe_3_C@C/CNF and Fe_3_C@C/CNF_AC_ materials, being 346±321 and 64±39 nm, respectively (Figure 1c, d).The less broader nanoparticle size distribution (i. e., 39 nm, Figure 1d) observed for Fe_3_C@C/CNF_AC_ indicates a more homogeneous composition compared to Fe_3_C@C/CNF that shows a wider size distribution (321 nm, Figure 1c) encompassing two types of nanoparticle populations.

For example, based on the degree of carbon protection three different types of nanoparticles can be found: big nanoparticles with an oxide shell (always bigger than ∼150 nm), nanoparticles with a very thin carbon shell (3 nm thickness corresponding to 8 graphitic layers with a d‐spacing of 0.35 nm), and relatively small nanoparticles coated with very thick carbon layers (Figure 1e–g). From high‐resolution TEM images, it was found that Fe_3_C@C/CNF contains unprotected and partially protected particles in similar proportions, while Fe_3_C@C/CNF_AC_ contains mostly well‐protected nanoparticles (with a few unprotected and partially protected ones). Inner core of all types of nanoparticles confirmed the interplanar distances of Fe_3_C, measured as 0.198 and 0.210 nm, corresponding to the characteristic crystalline planes (112) (2*θ*=45.9°) and (211) (2*θ*=60.8°) of Fe_3_C, respectively (Figure 1e, f). In contrast, an interplanar distance that corresponds to α‐Fe (011) plane (i. e.,=0.203 nm, Figure 1g) was measured in the fully protected particles of the Fe_3_C@C/CNF_AC_ hybrid material. Chemical mapping using energy dispersive X‐ray spectroscopy (EDS) in scanning transmission electron microscopy (STEM) was used to confirm the presence of iron, carbon and oxygen in the NPs (Figure S11).

Thus, acid‐treated CNF yield to smaller fully protected α‐Fe nanoparticles, covered with carbon graphitized layers, whereas untreated CNF yield to bigger Fe_3_C nanoparticles that are only partially protected. The observed difference in morphology and composition between Fe_3_C@C/CNF and Fe_3_C@C/CNF_AC_ suggests that CNF is not an innocent support, as the nanoparticle‐support interaction plays an important role during the annealing process.

**Figure 2:**
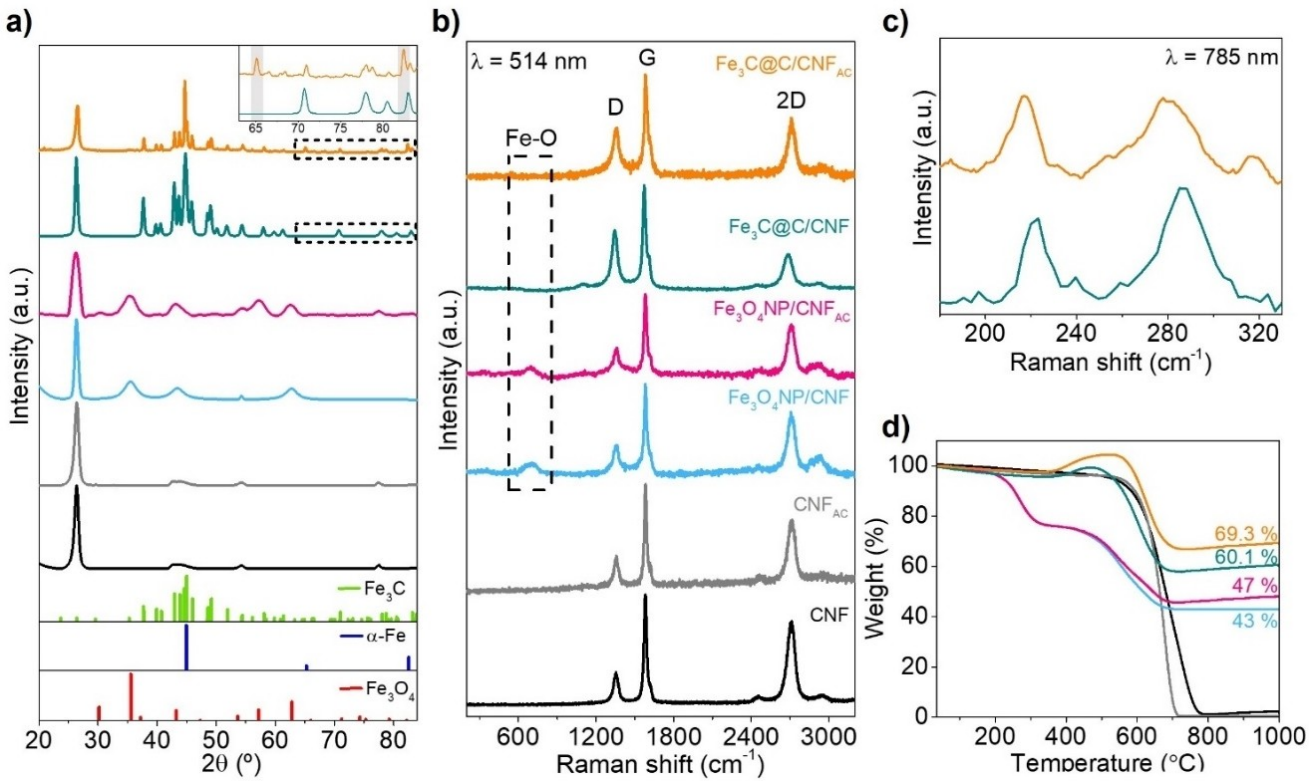
a) Powder XRD of samples before and after thermal treatment. CNF and CNF_AC_ controls are shown for comparison and Fe_3_C, α‐Fe and Fe_3_O_4_ as references. b) Raman spectra of all the samples before and after thermal treatment with a *λ*=514 nm laser. c) Raman spectra of Fe_3_C@C/CNF and Fe@Fe_3_C@C/CNF_AC_ using a *λ*=785 nm laser. d) TGA in air of samples before and after thermal treatment, with CNF and CNF_AC_ as controls.

X‐ray diffraction (XRD) reveals that the thermal treatment significantly changes the crystalline phases of iron nanoparticles. Hence, it is observed that the initial crystalline structure of Fe_3_O_4_NP (peaks located at 35.5° and 62.8°) is transformed to α‐Fe and Fe_3_C phases after thermal treatment (Figure 2a). Interestingly, the thermally produced materials present different compositions, further corroborating the picture that the nanoparticle‐support interaction templates the nanoparticle formation, as suggested by TEM measurements. Hence, Fe_3_C@C/CNF shows diffraction peaks that can only be assigned to Fe_3_C phase (and no other iron phases were identified). In contrast, for Fe_3_O_4_NP supported on acid‐treated CNF (i. e., Fe_3_C@C/CNF_AC_), a mixture of phases (Fe_3_C and α‐Fe) after thermal treatment can be noticed by the increase in the intensity of the peak at 45.0° (2θ) and the presence of two new 2θ peaks (65.3 and 82.4) that are not observed for Fe_3_C@C/CNF (inset in Figure 2a).

As shown in Figure 2b, Raman spectra of the hybrid composites before thermal treatment show characteristic Fe−O band at around 680 cm^−1^, along with the characteristic D, G and 2D carbon bands. In contrast, it is observed that the Fe−O band disappears in both composites after thermal treatment (Figure 2b, dashed rectangle) that is accompanied with a sharp increase in the I_D_/I_G_ ratios (from ∼0.33 to ∼0.52, Table S2). The increase in this ratio could be due to the defects produced by nanoparticles damaging the carbon structure during their diffusion and growth at high temperature. The formation of Fe_3_C after the thermal treatment was confirmed by the presence of two bands (i. e., 210 and 275 cm^−1^) by using a 785 nm laser (Figure 2c);^[28]^ this is in agreement with XRD results.

Thermogravimetric analysis (TGA) in air of the hybrid materials before thermal treatment (Figure 2d, pink and blue) reveal two weight losses related to the oxidation of organic surfactant (24.1 %, 270 °C) and carbon nanofibers (∼30 %, 570 °C), respectively. The solid residue remaining at 1000 °C in both samples is similar (42–48 % by weight), which clearly demonstrates that they contain similar amount of NPs. In contrast, thermally treated composites (Figure 2d, green and orange) exhibit no initial weight loss but a weight increase at 470 °C for Fe_3_C@C/CNF and at 520 °C for Fe_3_C@C/CNF_AC_, which is associated to the simultaneous oxidation of the carbon shell around the particles (observed in TEM images, Figure 1e–g) and the oxidation of iron (in Fe_3_C@C/CNF_AC_) and iron carbide core (in Fe_3_C@C/CNF). As expected, the higher weight increment in Fe_3_C@C/CNF_AC_ correlates well with TEM analysis and further confirms that larger number of protected iron nanoparticles (Figure 1g) are present in Fe_3_C@C/CNF_AC_ compared to Fe_3_C@C/CNF_._ After all the carbon burned up at 1000 °C, similar residual material of iron oxide (of 69.3 and 60.1 %, respectively) was observed for both thermally annealed samples.

**Figure 3:**
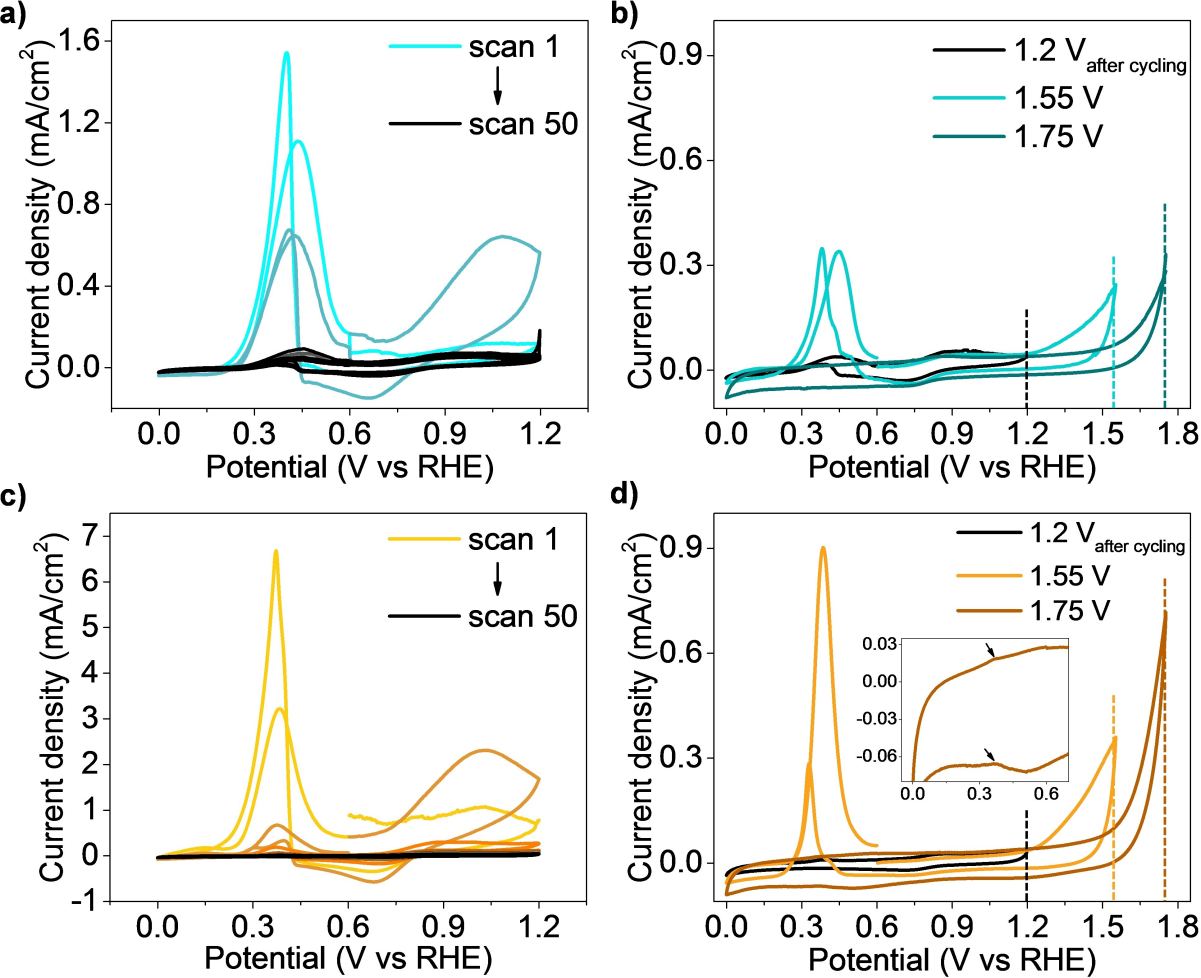
CV measurements of Fe_3_C@C/CNF a) during 50 CV cycles and b) after a stability test increasing the potential window, showing the appearance of a Fe_3_C redox peak. CV measurements of Fe_3_C@C/CNF_AC_ c) during 50 CV cycles and d) after a stability test increasing the potential window, showing the appearance of a Fe_3_C redox peak.

To further elucidate the encapsulation of Fe‐based NPs, both Fe_3_C@C/CNF and Fe_3_C@C/CNF_AC_ were electrochemically characterized in an acid electrolyte (0.1 M HClO_4_) in harsh oxidative conditions (i. e., high positive potential) in a three‐electrode set‐up. As shown in Figure 3, the sharp difference in current is in agreement with the expected higher surface area of Fe_3_C@C/CNF_AC,_ as it has much smaller nanoparticles than Fe_3_C@C/CNF based on TEM analysis. Moreover, both materials exhibit a similar redox behavior in a potential window from 0 to 1.2 V, which is related to the reversible Fe^2+^/Fe^3+^ redox process (between 0.7 and 1 V) and the complex oxidation of Fe_3_C to Fe^2+^ (between 0.2 and 0.5 V).[^29^, ^30^] Due to the irreversibility of oxidation of Fe_3_C to aqueous Fe^2+^, it can be observed that successive potential cycles lead to the dissolution of iron in the acid electrolyte as represented by the decrease in both Fe_3_C/Fe^2+^ and Fe^2+^/Fe^3+^ redox signal. The observation of Fe^2+^/Fe^3+^ redox process after 50 cycles in Fe_3_C@C/CNF agrees with the fact that large nanoparticles dissolve slower than small nanoparticles in Fe_3_C@C/CNF_AC_.

To evaluate the degree of carbon shell protection, graphitic carbon was electrochemically oxidized by applying a potential above 1.2 V.^[31]^ These electrochemical processes are shown in Scheme S2. Briefly, cyclic voltammetry (CV) measurements were further performed after stability in a larger potential window (i. e., upper potential of 1.55 and 1.75 V) to damage the carbon structure around the particles, exposing the initially protected iron carbide particles. Indeed, Figure 3b and d confirm that increasing the upper potential limit from 1.2 to 1.55 V, the carbon layers around the nanoparticles can be oxidized, enabling the access of the electrolyte to fresh Fe_3_C as suggested by the reappearance of Fe_3_C oxidation peak around 0.36 V. Note that the nanoparticles involved in this process are those for which their carbon shell is thick enough to protect them from atmospheric conditions, but thin enough to be able to oxidized them electrochemically. The sharper peak in Fe_3_C@C/CNF_AC_ (Figure 3d) compared to Fe_3_C@C/CNF (Figure 3b) further demonstrates that higher particle protection was achieved in Fe_3_C@C/CNF_AC_ due to the higher amount of iron nanoparticles protected by carbon layers. A further increase in the upper potential limit to 1.75 V reveals that some Fe_3_C is still protected in Fe_3_C@C/CNF_AC_ sample, but not in Fe_3_C@C/CNF (inset in Figure 3d). Thus, we evaluate the degree of encapsulation of metallic nanoparticles inside graphitic carbon by electrochemical means.

**Scheme 2:**
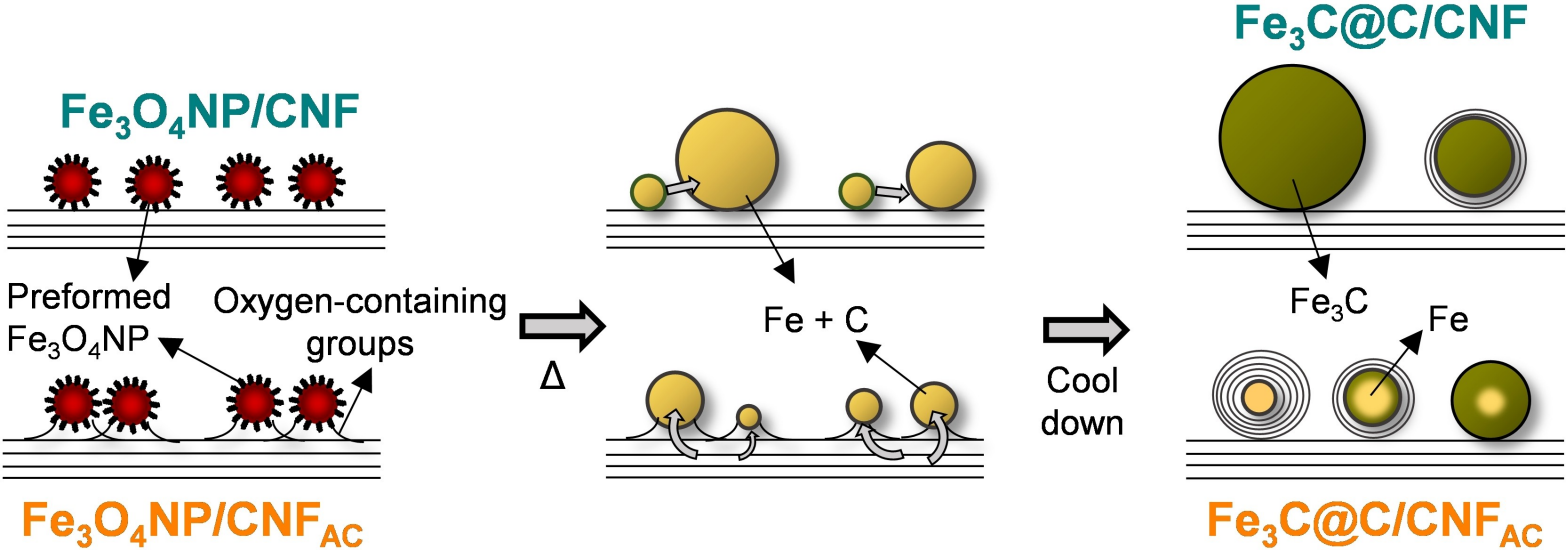
Schematic representation of the proposed mechanism for the transformation of Fe_3_O_4_NP/CNF precursors into Fe_3_C@C/CNF materials and the influence of the different carbon supports on the final morphology and composition of the nanoparticles.

All the above observations indicate that the size (small or large) and the chemical composition (iron carbide or metallic iron) of the final iron nanoparticles after thermal treatment are intimately dependent on the nanoparticle‐carbon support interaction. It is known that both structural (i. e., holes) and chemical (i. e., oxygen‐containing groups) defects are the most reactive sites on the carbon surface in CNF, so their presence would facilitate the dissolution of carbon on the pseudo‐liquid surface of iron nanoparticles.^[32]^ Additionally, it is expected a stronger attachment of iron nanoparticles to the surface of carbon nanofibers after the surface‐oxidation treatment based on previous studies.^[33]^ Thus, it is proposed that the presence of oxygen‐containing groups on the surface of CNF acts simultaneously as anchoring points preventing the nanoparticle agglomeration, and also as defects sites where carbon is prone to dissolve. Scheme 2 shows the process proposed for explaining the formation of different iron nanoparticles from the same nanoparticle precursor (i. e., Fe_3_O_4_NP, 4 nm) after thermal treatment. Briefly, the formation of iron‐carbon core‐shell nanoparticles is explained by the carbon dissolution‐precipitation model in which iron oxide is initially reduced to metallic iron by carbon gasification process.^[34]^ Then, the high temperature facilitates the dissolution of carbon into the melted iron nanoparticles, which undergo diffusion and growth processes such as Ostwald ripening and coalescence. However, these would be much hindered on nanoparticles supported on CNF_AC_ in contrast to CNF. As a result, the growth of the nanoparticles located on the surface of acid‐treated carbon nanofibers (CNF_AC_) would be limited and they would be capable of dissolving much more carbon than the large agglomerated nanoparticles in CNF, as the solubility of carbon in iron is intimately determined by the nanoparticle size (Scheme 2).^[35]^ The saturation, by which carbon precipitates into multilayer graphitic carbon layers around the iron nanoparticle, would be achieved for the small nanoparticles, whereas large particles, with much less carbon concentration, will lead mainly to the formation of carbide.^[36]^

**Figure 4:**
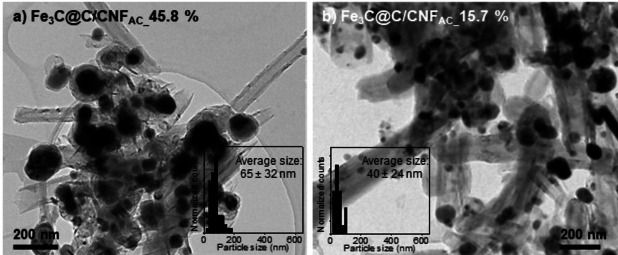
HRTEM images and size distribution of Fe_3_C@C/CNF_AC_ with iron contents of 45.8 and 15.7 %.

Overall, the introduction of acid groups on the carbon support precursors leads to a higher interaction with the preformed nanoparticles, and hence their diffusion is avoided, and a higher dissolution of carbon is promoted, producing materials with controlled particle size and composition. It is important to highlight that the iron content has an effect on the final morphology of the nanoparticle, but it is minimal in comparison to the effect of the nanoparticles‐support interaction. For example, it can be observed in Figure 4 that the decrease of the iron loading from 69 % to 45.8 % (Figure S12a) or to 15.7 % (Figure S12b) does not significantly change the nanoparticle morphology in the former case (i. e., neither in the nanoparticle size nor the carbon shell, Figure S13), while only slightly affects the nanoparticle size in the latter case. Thus, it is observed that the nanoparticle‐support interaction plays a much crucial role than the iron content to control the morphology of the nanoparticles.

### Evaluation of magnetic SAR for hyperthermia applications

To study the suitability of the produced hybrid materials for magnetic hyperthermia applications, magnetic SAR values, which correspond to the amount of energy that is transformed into heat per mass of magnetic material, were obtained using AC magnetometry. In comparison to conventional calorimetric methods, in which the increase in temperature of the sample is directly measured,^[37]^ the AC magnetometer used in this work allows faster data acquisition, high accuracy and determination of additional magnetic information.[^37^, ^38^] To test the materials under relevant conditions close to those of a real hyperthermia medical treatment, stable colloidal suspensions for both thermally treated materials (i. e., Fe_3_C@C/CNF and Fe_3_C@C/CNF_AC_) were prepared in water containing sodium dodecyl sulfate that is a biocompatible surfactant. By dynamic light scattering (DLS), the colloidal solution used for SAR measurements shows a monodisperse hydrodynamic radius (HR) of 2276 nm that remains practically unchanged after hours (Figure S14), highlighting its stability and suitability for practical applications.

**Figure 5:**
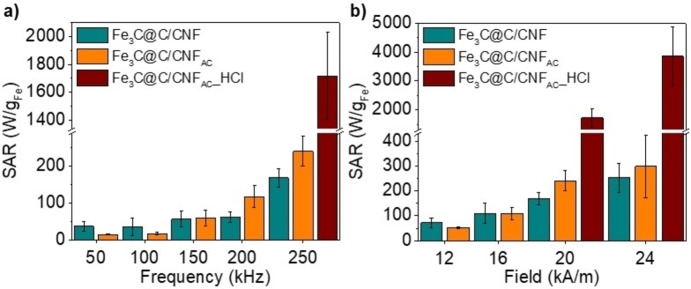
a) Frequency dependence of SAR values at a constant AC magnetic field of 20 kA m^−1^ and b) field dependence of SAR values at a constant frequency of 250 kHz of Fe_3_C@C/CNF and Fe_3_C@C/CNF_AC_ and Fe_3_C@C/CNF_AC_ after purification in HCl acid (Fe_3_C@C/CNF_AC__HCl).

Figure 5 shows the dependence of SAR with magnetic field frequency and intensity of both type of samples. Higher SAR values are measured for higher frequencies and intensities, showing a second order polynomial dependence. More importantly, it is shown that both materials are suitable for hyperthermia applications in medicine, as they can be used within the safety limit of the product of frequency by field amplitude (*f* × *H*), stated by Hergt as 5×10^9^ Am^−1^s^−1^.^[39]^

The higher SAR values of Fe_3_C@C/CNF_AC_ (i. e., 299 W g_Fe_^−1^ at 250 kHz and 24 kA m^−1^, Figure 5) in comparison to Fe_3_C@C/CNF (i. e., 253 W g_Fe_^−1^ at 250 kHz and 24 kA m^−1^, Figure 5) indicate the correlation between SAR and their structure. Its enhanced performance is due to the small particle size, which is related to a smaller amount of multidomain particles that could reduce the SAR efficiency, and the presence of α‐Fe phase in the core of the particles (Fe_3_C@C/CNF_AC_), since the magnetization of bulk α‐Fe (220 emu g^−1^) is higher than Fe_3_C (140 emu g^−1^).^[40]^

To reduce the negative effect of the larger oxidized nanoparticles, Fe_3_C@C/CNF_AC_ was purified by acid treatment in a HCl aqueous solution to dissolve the unprotected nanoparticles. The iron content of the resultant material (Fe_3_C@C/CNF_AC__HCl) decreased from 69.3 to 5.3 % as observed in TGA measurements (Figure S15), while CV and TEM measurements reveal that only small and protected particles remain in the material (Figure S16a–c). Statistical analysis of nanoparticle size shows very narrow distribution with a mean size of 54±21 nm (inset in Figure S16a). Magnetic SAR of purified Fe_3_C@C/CNF_AC_ (i. e., Fe_3_C@C/CNF_AC__HCl) reveals a significant increase (up to 3854 W g_Fe_^−1^ at the highest applied field, Figure 5b) due to the removal of large, unprotected and oxidized iron nanoparticles.

In comparison with other Fe/Fe_3_C based materials reported in literature, our hybrid nanostructure is found to have higher SAR values at lower intensities or frequencies (Table 1), which can be explained by the use of carbon nanofibers. In fact, we observed that carbon nanofibers, due to their high thermal conductivity, are able to enhance the SAR efficiency of preformed Fe_3_O_4_ NPs. Indeed, the SAR value of Fe_3_O_4_NP increases by a factor of ∼2 when they are supported on carbon nanofibers (i. e., Fe_3_O_4_NP/CNF) in comparison to free‐standing, as a consequence of the large area and high heat conductivity provided by the carbon nanofibers (Figure S17).^[28]^ Thus, the increase in thermal efficiency by using carbon nanofibers indicates the possibility of reducing the iron content, which is an ideal scenario to minimize the dose of nanoparticles to kill or deactivate the cancerous cells without damaging the healthy ones. Interestingly, the role of carbon nanofibers is not just restricted to the enhancement of the thermal conductivity of the composite, but also modifies the magnetic behavior of magnetic iron nanoparticles, as indicated by magnetic characterization. Hence, it is observed that Fe_3_O_4_NP exhibit, both as free‐standing and supported on carbon nanofibers, a transition from superparamagnetic to ferromagnetic by decreasing temperature below the blocking temperature as indicated by the in‐phase and out‐of‐phase signal of AC susceptibility at 1 Hz under zero DC field (Fe_3_O_4_NP in Figure S18 and Fe_3_O_4_NP/CNF in Figure S19). However, it is observed that the blocking temperature shifts to a significantly lower value from 61 to 16 K when Fe_3_O_4_ nanoparticles are supported on carbon nanofibers, being in agreement with the zero‐field‐cooled (ZFC)‐field cooled (FC) measurements with the field of 100 Oe in the temperature range 2–300 K (Figure S20).^[43]^ As no noticeably changes in nanoparticle size are observed by transmission electron microscopy after hybridization (Figure 1), this decrease of the blocking temperature can be undoubtedly assigned to the dispersion of the nanoparticles on the surface of carbon nanofibers.^[44]^ This is also in agreement with the observed variations of the blocking temperature within the 1–1000 Hz frequency range for Fe_3_O_4_ (Figure S18) and Fe_3_O_4_@CNF (Figure S19), thus indicating a typical behavior for interacting (Figure S21) and non‐interacting systems (Figure S22), respectively.^[45]^ Despite the decrease in blocking temperature, which is related to a decrease in the anisotropy and, hence, the SAR,^[46]^ magnetic saturation (another important parameter for SAR) of iron nanoparticles is not dramatically affected when combining with CNF which explain the overall improvement in the SAR capacity when Fe_3_O_4_ NPs are combined with CNF. Hence, magnetic hysteresis loops for Fe_3_O_4_ and Fe_3_O_4_NP/CNF further confirm that the nanoparticles are ferromagnetic below the blocking temperature (i. e., 2 K, Figure S23a) and superparamagnetic above the blocking temperature (i. e., 300 K, Figure S23b), as revealed by the absence of coercivity and remanence in the hysteresis loop at 300 K.^[47]^

**Table 1:** Comparison of SAR values reported in literature for Fe/Fe_3_C based materials.

Material	Conc. [mg mL^−1^]	Field [kA m^−1^]	Frequency [kHz]	SAR [W g_Fe_^−1^]	Ref.
Fe@CNT	5	100	139	1879	[25]
Fe@C	5	80	128	240	[41]
Fe/Fe_3_C@CNT bioconjugate	0.125	45	220	500	[24]
Fe/Fe_3_C@C	–	26	477	70.5	[40]
Fe_3_C@Pluronic acid F‐127	5	20	261	46	[42]
Fe@CNT	0.08	20	230	100	[20]
Fe_3_C@C/CNF	0.125	20	250	168	this work
Fe_3_C@C/CNF_AC_	0.125	20	250	241	this work
Fe_3_C@C/CNF_AC_ HCl	0.125	20	250	1716	this work

## Conclusion

A well‐protected reduced form of iron nanoparticles has been prepared at high temperatures by simply controlling the interaction of Fe_3_O_4_ NP precursors with the carbon support. Hence, it has been demonstrated that a high number of defects on the CNF support limits the growth of reduced iron nanoparticles and facilitates carbon dissolution during thermal treatment in a vacuum, leading to the formation of small and well‐protected α‐Fe‐based nanoparticles (Fe_3_C@C/CNF_AC_). In contrast, untreated CNF produce bigger nanoparticles, pure Fe_3_C phase, and less protected particles (Fe_3_C@C/CNF). The iron‐based hybrids obtained were evaluated for application in magnetic hyperthermia (MH) by using an AC magnetometer. The specific absorption rate (SAR) was calculated from hysteresis losses. The well‐protected α‐Fe‐based nanoparticles (Fe_3_C@C/CNF_AC_, 299 W g_Fe_^−1^) show a higher SAR value than unprotected iron nanoparticles (Fe_3_C@C/CNF, 253 W g_Fe_^−1^), thus confirming the possibility of SAR enhancement by an effective interaction between the carbon support and the iron nanoparticles. This was further demonstrated by the significant increase in SAR value of the material after purification with removal of the unprotected nanoparticles by using HCl. This work demonstrates a controlled design of new iron‐based confined nanostructures with high magnetic heating efficiency, which may be used for further devices in magnetic hyperthermia.

## Experimental Section

**Chemicals**: PR‐24‐XT‐HHT‐AM (PR24) carbon nanofibers were purchased from Pyrograph Products by Applied Sciences. All chemicals were purchased from Sigma–Aldrich and were used without further purification.

**Synthesis of iron oxide nanoparticles (Fe_3_O_4_NP)**: Fe_3_O_4_NP were synthesized according to the procedure published by Sun et al.^[27]^. A three‐neck flask was loaded with 2 mmol of iron(III) acetylacetonate, 10 mmol of hexadecane‐1,2‐diol, 6 mmol of oleic acid and 6 mmol of oleylamine in 20 mL of diphenyl ether and the mixture was stirred under argon flow. It was heated at an approximate rate of 5 °C min^−1^ to the final temperature of 250–260 °C, where it stayed refluxing for 30 min. Finally, it was cooled down and the nanoparticles were precipitated by adding ethanol and separated by centrifugation (using a ThermoFischer Heraeus Megafuge 8 with a MicroClick 24×2 rotor at 6000 rpm for 10 min).

**Milling of carbon nanofibers (CNF)**: 50 mg of PR24 were milled for 180 min at a frequency of 10 Hz in a mixer mill MM400.

**Acid treatment of milled carbon nanofibers (CNF_AC_)**: 50 mg of milled carbon nanofibers (PR24) were treated with 25 mL of 6 M HNO_3_ for 2 h at reflux in air (100 °C). After that, it was cooled down to room temperature and the nanofibers were collected using a hydrophilic 0.45 μm PTFE membrane. They were washed with distilled water until pH 7 and dried on the filter.

**Preparation of iron carbide particles encapsulated within graphitic carbon layers supported on modified carbon nanofibers (Fe_3_C@C/CNF)**: First, preformed iron oxide nanoparticles were combined with modified carbon nanofibers. A round flask was loaded with a mixture of iron oxide nanoparticles (60 mg) and carbon nanofibers (30 mg). It was suspended in hexane (30 mL) and sonicated for 1 min. The solvent was evaporated in a rotary evaporator at room temperature. The process was repeated 5 times. After that, the product was suspended one last time in hexane by 1 min sonication and filtered using a hydrophobic 0.2 μm PTFE membrane. The nanoparticles in excess were removed by washing the product with hexane on the filter. To evaluate the effect of the iron content, two samples with different Fe_3_O_4_NP:CNF_AC_ ratio (i. e., 1 : 1, 1 : 2) were prepared. The obtained hybrid nanostructures were then heated at 1000 °C for 3 h inside quartz tubes sealed in vacuum, each tube containing 30 mg of sample. After that time, they were taken out of the oven and left to cool down to room temperature.

**Purification of Fe_3_C@C/CNF_AC_ in HCl (Fe_3_C@C/CNF_AC_** _**HCl)**: Approximately 10 mg of thermally treated hybrid with acid carbon nanofibers (Fe_3_C@C/CNF_AC_) were mixed in 20 mL of HCl (37 %) in a round flask and refluxed in air (100 °C) for 4 h. After the reaction, the mixture was cooled down to room temperature and the sample was collected by vacuum filtration using a hydrophilic 0.45 μm PTFE membrane. Finally, it was washed with distilled water until neutral pH and left to dry.

**Characterization of the hybrid materials**: Thermogravimetric analysis were performed using a Mettler Toledo TGA/DSC 3+. The thermal program consisted of an isotherm at 30 °C for 10 min followed by a heating ramp of 5 °C min^−1^ until 1000 °C. A gas flow of 90 mL min^−1^ of air was employed as oxidant atmosphere. As reducing atmosphere, gas flow of 25 mL min^−1^ of nitrogen was applied. IR spectra were obtained with a universal attenuated total reflectance FTIR spectroscope (UATR Two PerkinElmer). The measurements were conducted in a spectral range from 400 to 4000 cm^−1^. A confocal Raman “Invia Reflex” was employed, with excitation lasers at 514 nm to observe carbon signals and at 785 nm for iron carbide signals. Samples were prepared by depositing a drop of a dispersion of the materials in hexane (∼1 mg mL^−1^) onto a microscope glass slide. TEM images were obtained with a field emission transmission electron microscope JEOL JEM‐F200 at an accelerating voltage of 200 kV. Samples were dispersed in hexane and drop casted onto holey carbon film on copper grids. The powder XRD patterns were obtained using a Bruker D8 Advance diffractometer equipped with a Cu_Kα_ radiation source (*λ*=1.5418 Å) operating at 40 kV and 40 mA and a LYNXEYE XE‐T detector. Magnetic measurements on Fe_3_O_4_ nanoparticles and Fe_3_O_4_@CNF hybrid were carried out in a commercial Quantum Desing MPMS‐XL5 Superconducting Quantum Interference Device (SQUID) magnetometer. Fe_3_O_4_ NP and Fe_3_O_4_@CNF samples were carefully prepared using a plastic capsule with a negligible diamagnetic contribution. The electrochemical behavior of the hybrid nanostructures was measured using a potentiostat–galvanostat PGSTAT302 N Autolab. Each sample was suspended in hexane (0.5 mg in 0.5 mL) by 5‐min sonication, and 60 μL were drop cast on a clean glassy carbon electrode (5 mm diameter). The experiments were carried out in 100 mL of 0.1 M HClO_4_ electrolyte solution under nitrogen by using a reversible hydrogen electrode (RHE) as reference and a graphite rod as counter electrode. A scan rate of 50 mV s^−1^ was applied for cyclic voltammetry. AC magnetometry measurements were carried out by a Combi AC Hyster Series from Nanotech Solutions in the frequency range from 50 to 250 kHz and intensity range from 8 to 24 kA m^−1^. Liquid dispersions were prepared by sonication of 1 mg of material in 1 mL of 0.1 wt% SDS aqueous solution, then dilution of this dispersion to prepare 0.125 mg of material per mL of 0.1 wt% SDS solution. The specific absorption rate (SAR) values were calculated according to SAR=A⋅f, where A is the magnetic area and f is the AC magnetic field frequency. SAR values are mass normalized using the iron loading obtained from TGA measurements.

## Conflict of interest

The authors declare no conflict of interest.

1

## Supporting information

As a service to our authors and readers, this journal provides supporting information supplied by the authors. Such materials are peer reviewed and may be re‐organized for online delivery, but are not copy‐edited or typeset. Technical support issues arising from supporting information (other than missing files) should be addressed to the authors.

Supporting Information

## Data Availability

The data that support the findings of this study are available from the corresponding author upon reasonable request.
