# Supplementary material for: Magnetic Hyperthermia Enhancement in Iron‐based Materials Driven by Carbon Support Interactions
Source: Chemistry. 2022 Oct 6;28(67):e202201861. doi: 10.1002/chem.202201861 (PMC10092447; doi:10.1002/chem.202201861)
Supplement: Supplementary file 1 — Supporting Information [file CHEM-28-0-s001.pdf]

Chemistry–A European Journal

Supporting Information

Magnetic Hyperthermia Enhancement in Iron-based Materials Driven by Carbon Support Interactions

Lucía Vizcaíno-Anaya, Carlos Herreros-Lucas, José M. Vila-Fungueiriño, and María del Carmen Giménez-López*

Index

	Page
1. Synthesis of preformed iron oxide nanoparticles	1
2. Structural and/or chemical modification of carbon nanofibers prior to combine with preformed Fe_3O_4 nanoparticles	4
3. Materials before and after thermal treatment	7
4. Electrochemical study of Fe_3C@C/CNF materials	10
5. Role of iron content in the morphology of Fe_3C@C/CNF_{AC}	12
6. Colloidal stability of Fe_3C@C/CNF_{AC}	13
7. Purification of Fe_3C@C/CNF_{AC} with HCl (Fe_3C@C/CNF_{AC}_HCl)	14
8. Magnetic properties of Fe_3O_4NP and Fe_3O_4NP/CNF	15
9. References	19

1. Synthesis of preformed iron oxide nanoparticles

Iron oxide nanoparticles (Fe_3O_4NP) were synthesized following a procedure reported by Sun *et al.*[1] in which iron(III) acetylacetonate ($Fe(acac)_3$) was mixed with 1,2-hexadecanediol as reducing agent, oleic acid and oleylamine as surfactants in diphenyl ether and heated under argon atmosphere (**Scheme S1**). In the resultant mixture, Fe^{3+} ions were partially reduced by the alcohol reagent and formed Fe_3O_4 nanoparticles stabilized by surfactant molecules.

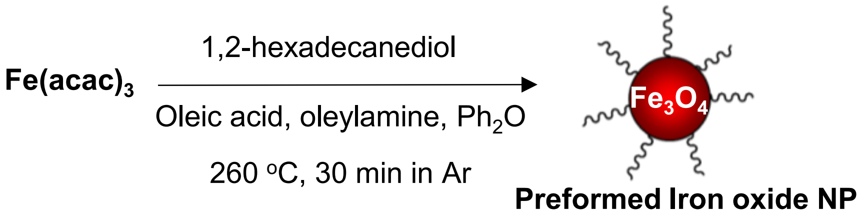

Scheme S1. Schematic representation of the synthetic procedure followed for the preparation of preformed Fe_3O_4 nanoparticles.

HRTEM showed a narrow size distribution and a mean diameter of Fe_3O_4 nanoparticles in 3.7 ± 0.6 nm **(Figure S1a, b)**.

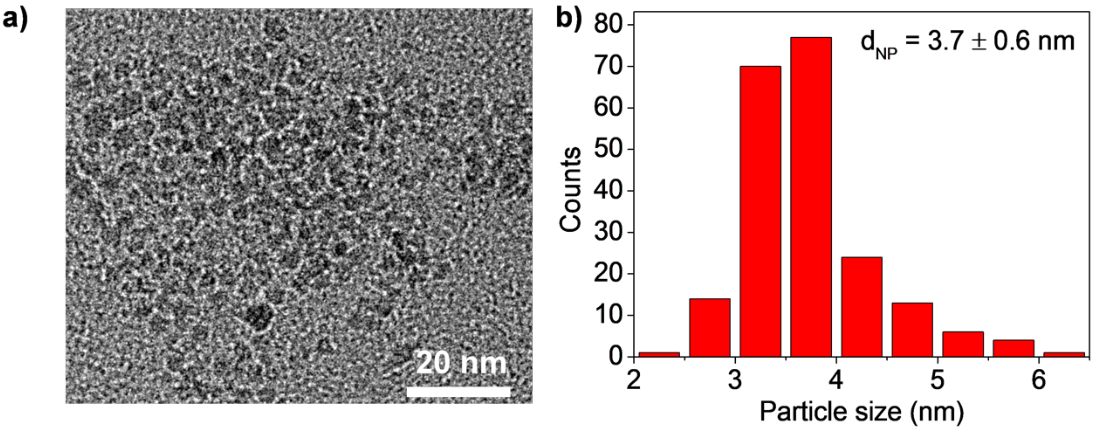

Figure S1. (a) HRTEM image of preformed nanoparticles. **(b)** Size distribution of preformed nanoparticles, indicating the mean diameter.

IR and Raman spectroscopy were used to determine the chemical composition of the Fe_3O_4 nanoparticles with surfactant**.** The IR spectra of Fe_3O_4 nanoparticles demonstrated the bands corresponding to functional groups of the surfactant molecules, asymmetric and symmetric COO^- stretching bands at 1420 and 1525 cm^{-1}, and a C-O stretching band at 1050 cm^{-1} (**Figure S2a**).[2] Two sharp bands at 2850 and 2920 cm^{-1} can be attributed to the symmetric and asymmetric CH_2 stretches, respectively. The presence of a sharp IR band at 570 and a shoulder at 700 cm^{-1} and the characteristic absorption peaks of the Fe-O vibrations are related to Fe_3O_4.[3] Raman spectra of Fe_3O_4 revealed a strong band at 670 cm^{-1} which was assigned to the A_{1g} mode, along with bands at 306 and 538 cm^{-1}, corresponding with E_g and T_{2g} modes, respectively (**Figure S2b**). It was also observed that Fe_3O_4 nanoparticles indicate a systematic

shift of ~35 cm^{-1} compared to reported Fe_3O_4 bands, which has been attributed to defects and partial disorder.[4]

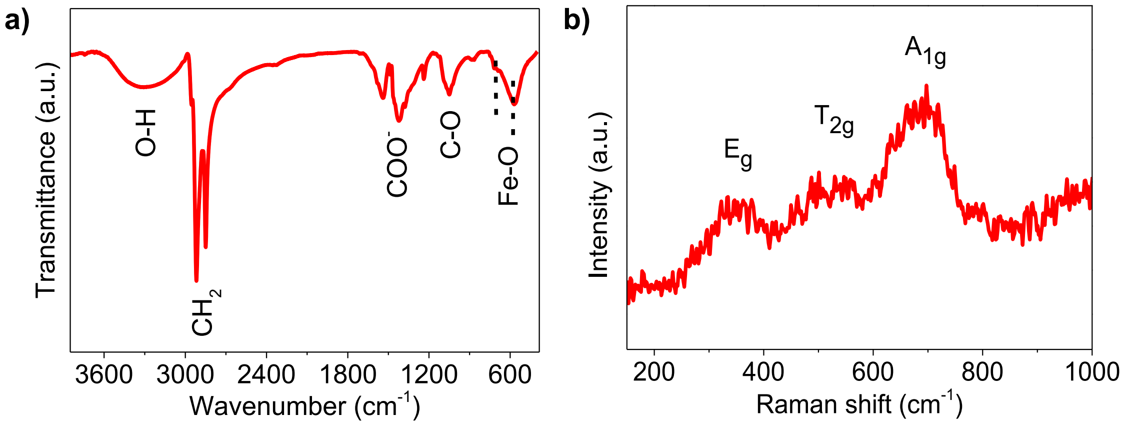

Figure S2. (a) Infrared spectra of preformed nanoparticles, indicating the nature of each band. **(b)** Raman spectrum of preformed nanoparticles, indicating the Raman modes of each band.

Powder XRD was performed to study the crystallinity of iron oxide nanoparticles and compared them with the standard reference patterns of Fe_3O_4 and Fe_2O_3 (**Figure S3**). Peaks observed at 35.4, 56.9 and 62.5 2θ degrees correspond to the lattice planes of (311), (511) and (440), respectively, for the cubic structure of Fe_3O_4 (Magnetite).[5] The particle size can also be estimated using Debye-Scherrer equation via XRD (**eq. S1**). It was calculated that Fe_3O_4 nanoparticles have a mean diameter of ~4 nm which was in accordance with the size measured via HRTEM **(Figure S1b).**

- Calculation of average particle size (τ) from XRD data using Debye-Scherrer equation (**eq. S1**):

$$\tau = \frac{K\,\lambda}{\beta\,cos\theta} = \frac{0.89\cdot 1.5418\ \text{Å}}{0.034\cdot\cos 17.7} = 42.2\ \text{Å} = 4.22\ nm \qquad \textbf{(eq. S1)}$$

where K is a shape factor with value 0.89 for spherical particles, λ is the X-ray wavelength (1.5418 Å for CuK_α), β is the line broadening at half the maximum intensity in radians and θ is the diffraction angle.[5]

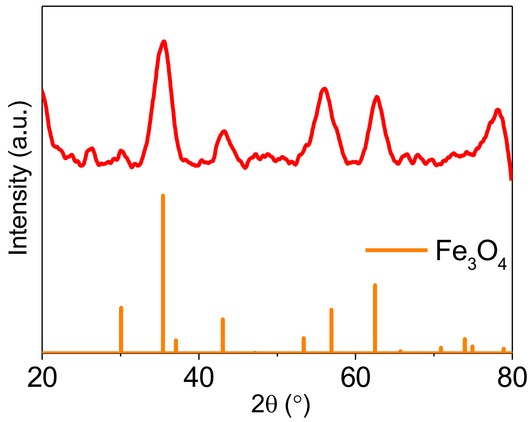

Figure S3. X-ray diffraction spectra of preformed nanoparticles, compared with standard Fe_3O_4 pattern.

TGA was used to study the thermal behaviour and the surfactant/iron oxide mass ratio of Fe_3O_4 between the room temperature and 1000 °C in air atmosphere (**Figure S4**). The weight loss between 200 and 300 °C corresponds to the surfactant measured as 36.6 % by weight.[5,6]

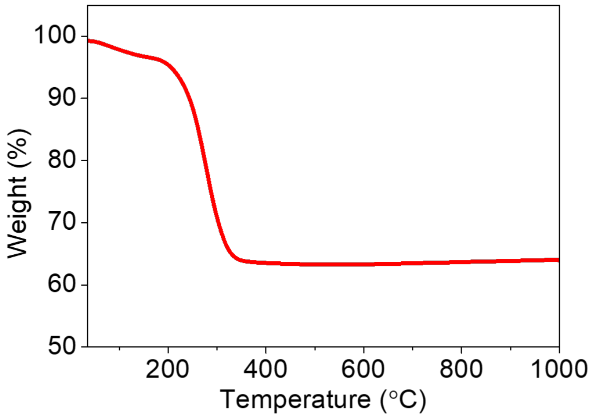

Figure S4. Thermogravimetric analysis curve of preformed iron oxide nanoparticles.

2. Structural and/or chemical modification of carbon nanofibers prior to combine with preformed Fe_3O_4 nanoparticles

Commercially available carbon nanofibers possess a diameter of ~100 nm, wall thickness of 28 ± 6 nm, and a length of ~10 μm. They consist of externally a number of graphitic layers while internally corrugated step-edges with a few carbon layers behaving like anchoring points to interact and stabilise a number of different molecules and/or nanoparticles.[7] However, these fibers are very hydrophobic and it is required a number of chemical and physical modifications prior to use them in solution phase reactions such as creating functional groups/defects and/or shortening them.

Mechanical ball milling has recently been used to shorten carbon nanofibers to eliminate the mass transportation problems in solutions. Acid treatment is also commonly used to oxidize the carbon nanomaterials by producing acid functional groups. Overall, both ball milling and acid treatment provide attachment sites for the nanoparticles, as it has been proven in literature.[8,9]

Carbon nanofibers were ball milled for 180 minutes at a frequency of 10 Hz using a mixer mill MM400. Then, some of those milled nanofibers were acid treated in refluxing nitric acid for 2 hours to obtain a more oxidized carbon support from the combination of the different defects produced in each treatment. They are referred to as CNF and CNF_{AC}, respectively.

CNF and CNF_{AC} were characterized via TGA both under inert and air atmosphere to quantify the functional groups created and to determine the oxidation temperature of these materials (**Figure S5**). As expected, CNF_{AC} exhibited a higher weight loss between around 100 and 400 °C compared to CNF (3 % compared to 2.5 %) under inert atmosphere. However, both types of fibers show very similar behaviour**.**

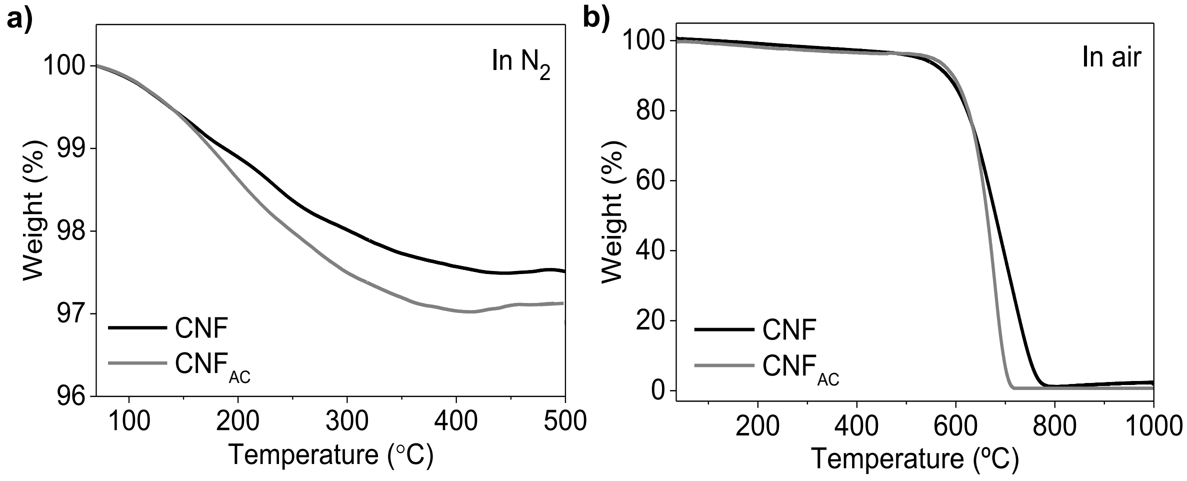

Figure S5. (a) TGA under nitrogen atmosphere of modified carbon nanofibers CNF and CNF_{AC}. **(b)** TGA in air of CNF and CNF_{AC}.

Raman spectroscopy demonstrated the presence of the characteristic carbon bands for each material: D band corresponds to the presence of sp^3 carbon (located at 1350 cm^{-1}), G band represents the graphitic carbon (located at 1580 cm^{-1}) and 2D band is an indicative of high ordered nanographites (located at 2710 cm^{-1}). The ratio between intensities of D and G band (I_D/I_G) is used to quantify the degree of defects on the carbon structure.[10] This confirmed that

CNF_{AC} has higher number of defects with a I_D/I_G ratio of 0.351 compared to CNF (I_D/I_G = 0.298) (**Figure S6 and Table S1**).

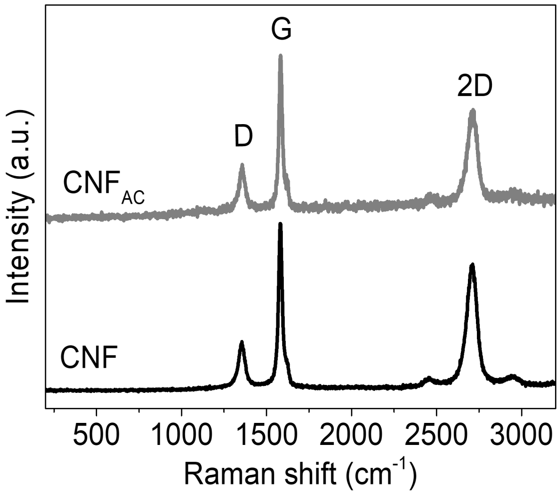

Figure S6. Raman spectra at 514 nm showing the characteristic carbon bands.

Table S1. Degree of defects in the modified carbon nanofibers, quantified by the Raman spectroscopy I_D/I_G ratios.

Sample	I_D/I_G ratio
CNF	0.298
CNF_{AC}	0.351

Comparing XRD spectra, CNF and CNF_{AC} have very similar patterns and graphite characteristic peak can be observed at 2θ = 26.6°, corresponding with crystalline plane (002). Other peaks are found with low intensity at 2θ = 44.3° (101), 54.5° (004), 77.5° (110) and 83.5° (112) (**Figure S7**).

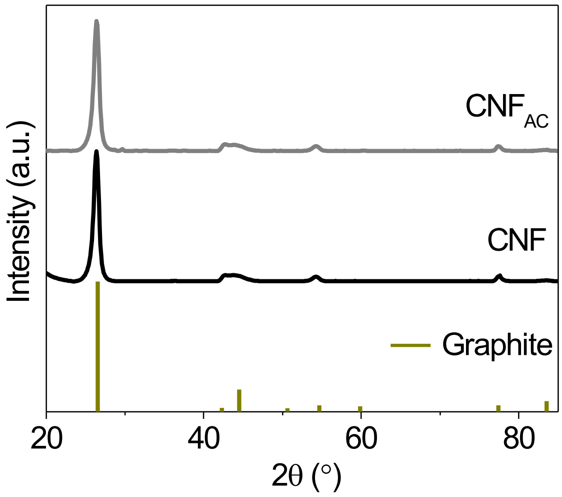

Figure S7. XRD spectra of CNF and CNF_{AC} compared with standard graphite pattern.

Finally, cyclic voltammetry was performed to observe the redox behaviour of CNF and CNF_{AC} (**Figure S8**). The introduction of oxygen containing functional groups in CNF and CNF_{AC} is confirmed by the characteristic redox process of surface quinoidal functional groups observed at 0.63 V vs RHE, which was slightly more characteristic in CNF_{AC} as expected (**eq. S2**).[11]

$$C_{aromatic}=O + H^{+} + e^{-} \rightleftharpoons C\text{-}O\text{-}H \qquad E^{o} = 0.63V \text{ vs RHE} \quad (\textbf{eq. S2})$$

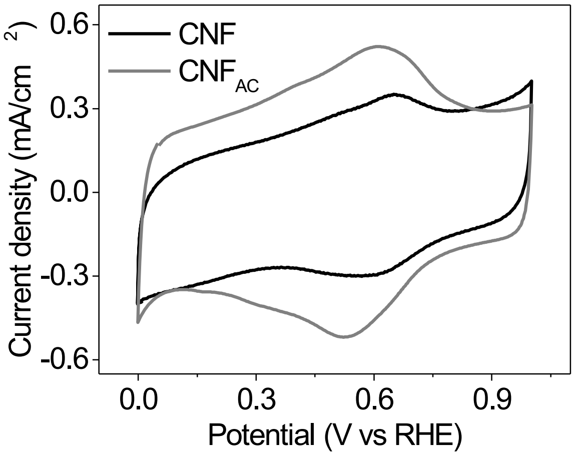

Figure S8. Cyclic voltammograms of CNF and CNF_{AC} in 0.1M $HClO_4$.

By these treatments, we obtained materials that, even though they have very similar characteristics, slightly different types of defects on the structure enable stronger interactions with nanoparticles.

3. Materials before and after thermal treatment

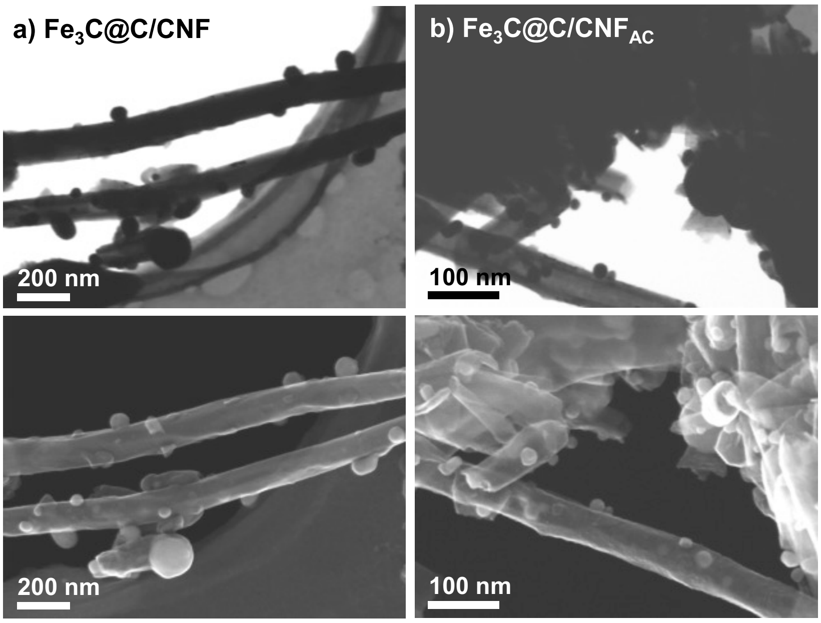

Figure S9. Bright and dark field STEM images of the materials after thermal treatment with **(a)** CNF, and **(b)** CNF_{AC}.

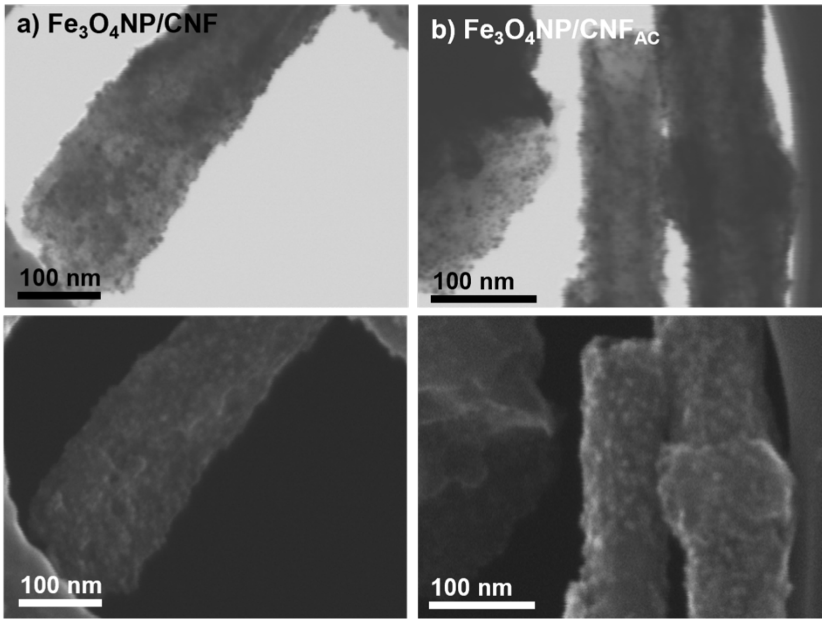

Figure S10. Bright and dark field STEM images of the materials before thermal treatment with **(a)** CNF, and **(b)** CNF_{AC}.

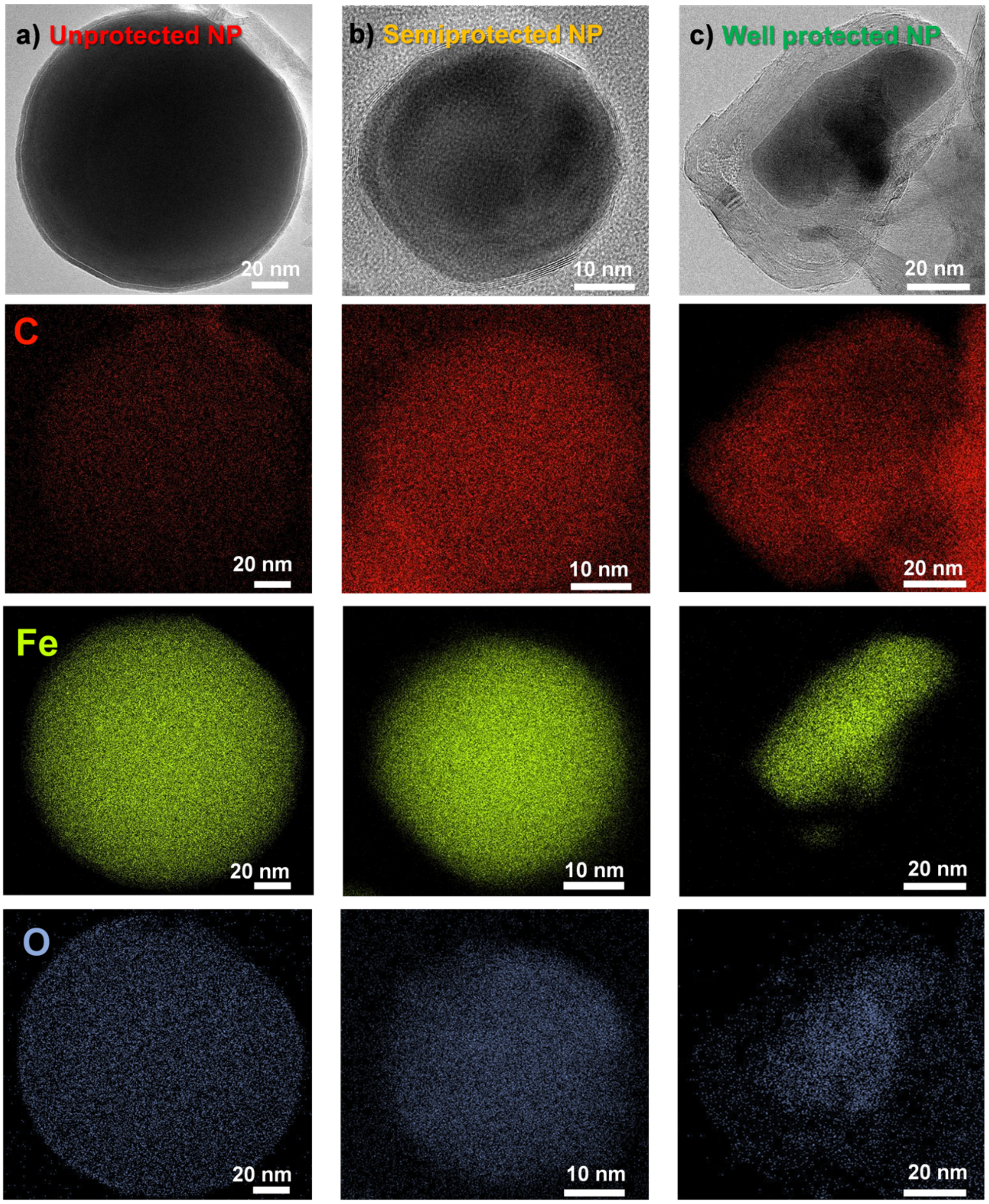

Figure S11. HRTEM images and EDS mapping of three main types of Fe_3C nanoparticles found on the carbon nanofibers.

Table S2. Degree of defects in the modified carbon nanofibers of the hybrid materials before and after the thermal treatment, quantified by the Raman spectroscopy I_D/I_G ratios observed in Figure 2b.

Sample	I_D/I_G ratio, before thermal treatment	I_D/I_G ratio, after thermal treatment
Fe_3C@C/CNF	0.329	0.593
Fe_3C@C/CNF_{AC}	0.342	0.524

4. Electrochemical study of Fe_3C@C/CNF materials

The electrochemical processes involved in the oxidation of protective carbon layers and exposure of Fe_3C particles were studied and the mechanism is represented in **Scheme S2**.

After 50 CV cycles between 0 and 1.2 V, no redox behavior of iron is observed, confirming that all unprotected nanoparticles were dissolved. The following CV cycle performed in a wider potential window between 0 to 1.55 V demonstrated that once the potential reaches 1.55 V in onward scan, the carbon layer is oxidised and broken, enabling the fresh Fe_3C outermost of the nanoparticle core to be exposed into acid (**A in Scheme S2**). In the backward scan, this fresh material is oxidized and Fe_3C oxidation peak was observed at 0.36 V (**eq. S3 and B in Scheme S2**). The contact of fresh iron/iron carbide with the acid electrolyte generates an iron oxide shell around the particles (passivation), which explains the reappearance of the redox signal at 0.85 V, corresponding with **eq. S4** (**C in Scheme S2**). Fe^{3+} ions are easily dissolved in the electrolyte solution and the nanoparticles lose mass during following cycles until they disappear.

$$\mathrm{Fe_3C \rightarrow 3\ Fe^{2+} + C + 6\ e^-} \qquad \textbf{(eq. S3)}^{[12,13]}$$

$$\mathrm{Fe^{2+} \rightleftharpoons Fe^{3+} + 1\ e^-} \qquad \textbf{(eq. S4)}$$

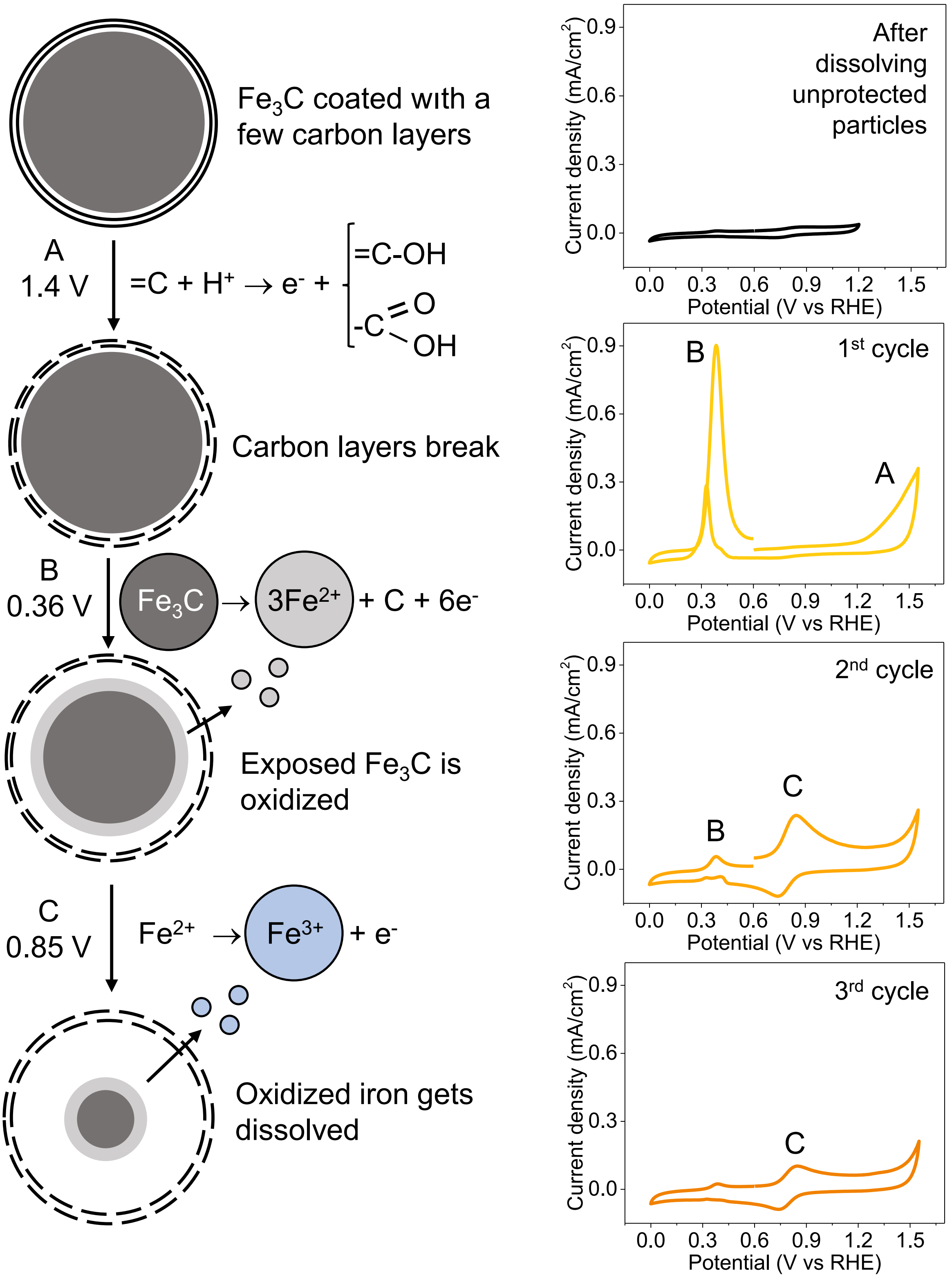

Scheme S2. Proposed mechanism showing the iron dissolution on the nanoparticles coated with thin carbon layer by continuous CV.

5. Role of iron content in the morphology of $Fe_3C@C/CNF_{AC}$

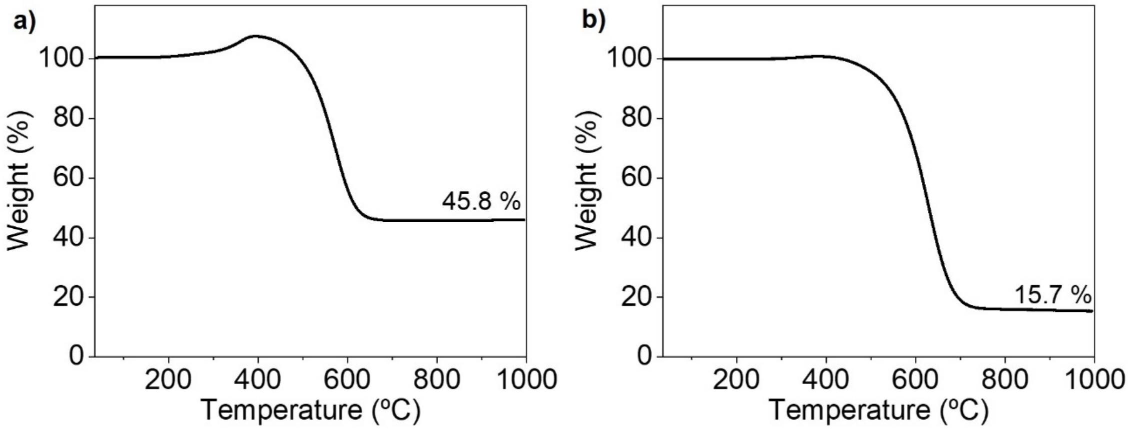

Figure S12. TGA of $Fe_3C@C/CNF_{AC}$ with different iron content (45.8 and 15.7%, respectively) in air.

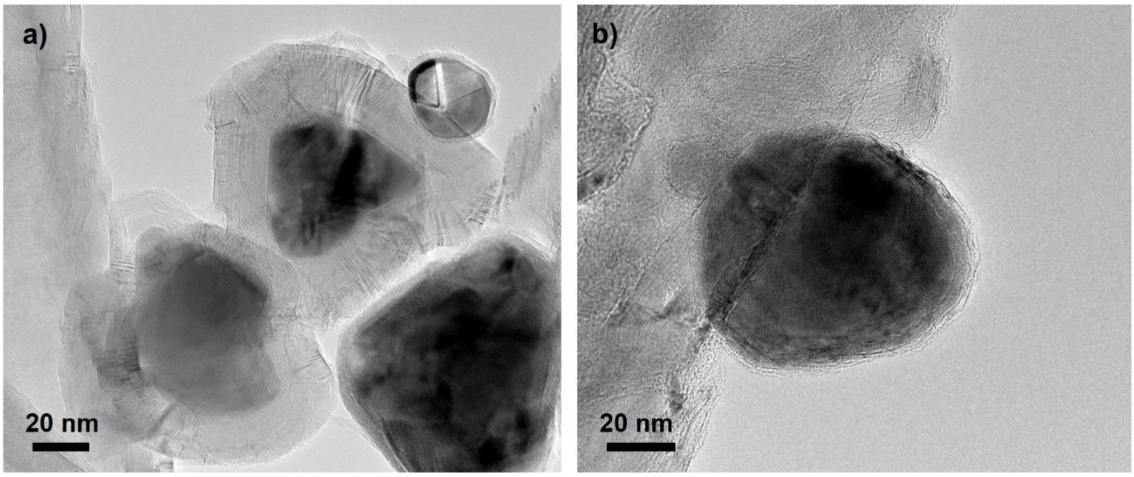

Figure S13. HRTEM images of $Fe_3C@C/CNF_{AC}$ with small iron content (45.8 and 15.7%, respectively).

6. Colloidal stability of Fe_3C@C/CNF_{AC}

The stability of the colloidal solution made of Fe_3C@C/CNF_{AC} in a 0.1 wt% SDS aqueous solution was evaluated by measuring the changes in the hydrodynamic radius (HR) obtained by DLS when the suspension is left self-standing for 60 minutes after sonication.

As observed in **Figure S14**, the HR of the sample immediately after sonication (initial) is 2276 nm. The sample shows great colloidal stability, with a slight decrease in HR to 1900 nm after 60 min, being suitable for practical applications.[14] Additionally, DLS was performed after 16 h and the HR only exhibited a small decrease.

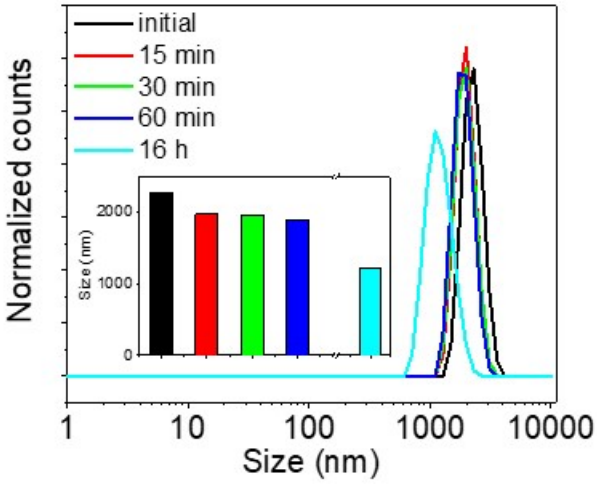

Figure S14. DLS measurements of the aqueous suspension of Fe_3C@C/CNF_{AC} used in the hyperthermia experiments (0.125 mg in 1 mL of 0.1 wt% SDS solution) after 15, 30, 60 min and 16 hours. The initial sample was previously sonicated for 10 minutes and then was left self-standing to monitor its stability with DLS. The hydrodynamic radious of the sample stayed within the range 1.8 to 2.2 µm after 60 min.

7. Purification of $Fe_3C@C/CNF_{AC}$ with HCl ($Fe_3C@C/CNF_{AC}$_HCl)

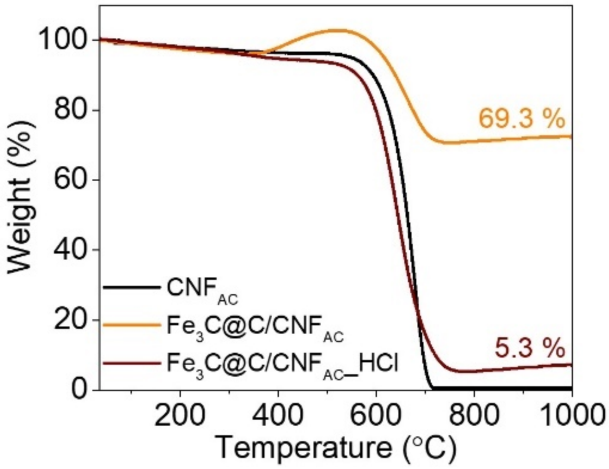

Figure S15. TGA of $Fe_3C@C/CNF_{AC}$ after being purified with HCl ($Fe_3C@C/CNF_{AC}$_HCl), showing a decrease in the iron content (from 69.3 to 5.3%).

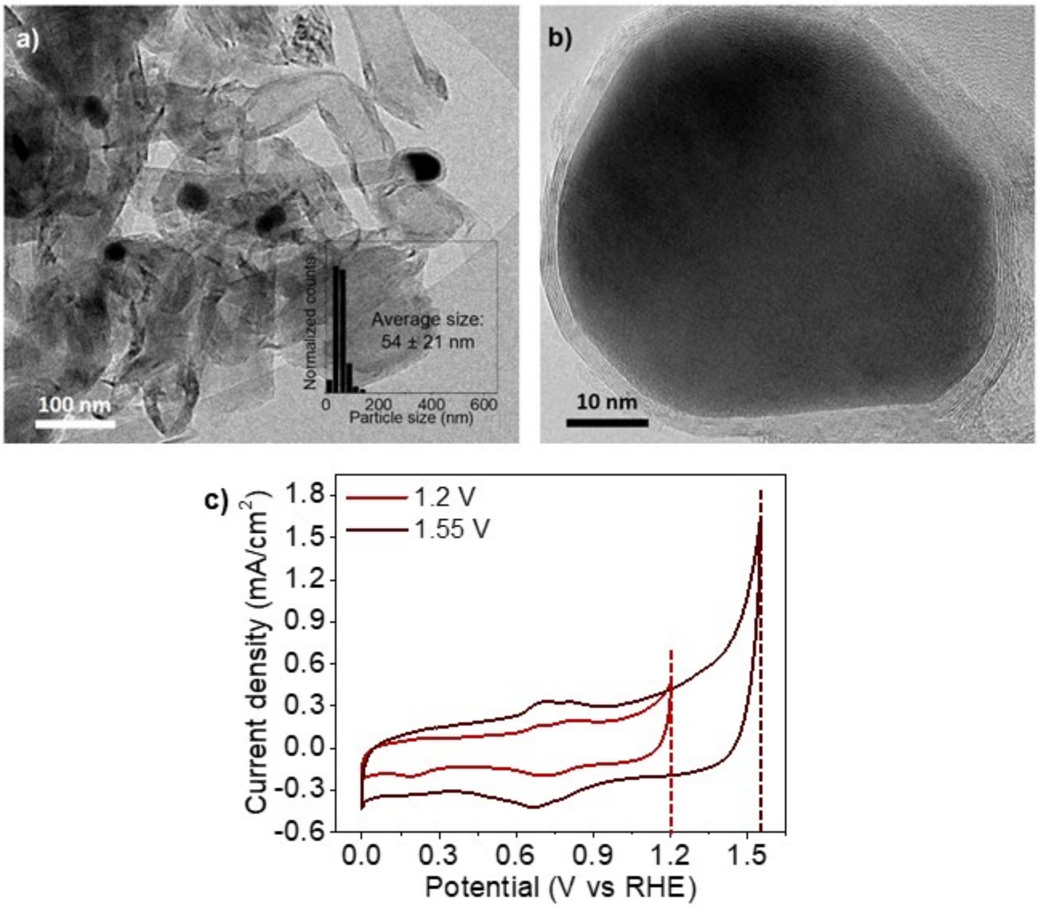

Figure S16. a-b) HRTEM images and size distribution of $Fe_3C@C/CNF_{AC}$ after being purified with HCl ($Fe_3C@C/CNF_{AC}$_HCl). c) CV measurements for $Fe_3C@C/CNF_{AC}$_HCl demonstrating the absence of unprotected nanoparticles.

8. Magnetic properties of Fe_3O_4NP and Fe_3O_4NP/CNF

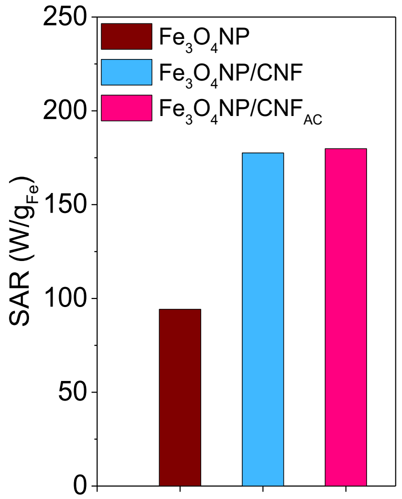

Figure S17. SAR values of free-standing nanoparticles (94.2 W/g_{Fe}) and nanoparticles supported on CNF (177.6 W/g_{Fe}) and CNF_{AC} (179.8 W/g_{Fe}), measured at 250 kHz and 20 kA/m.

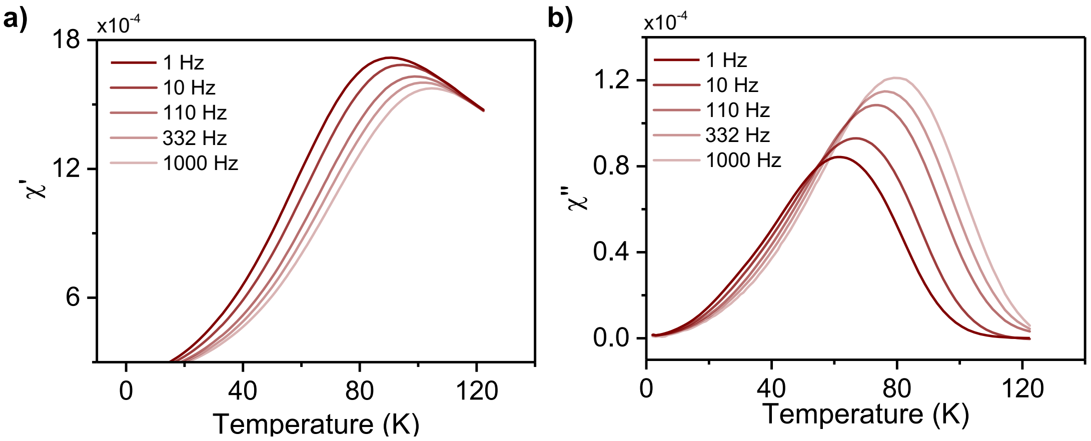

Figure S18. **(a)** In-phase and **(b)** out-of-phase susceptibility at different frequencies of Fe_3O_4NP.

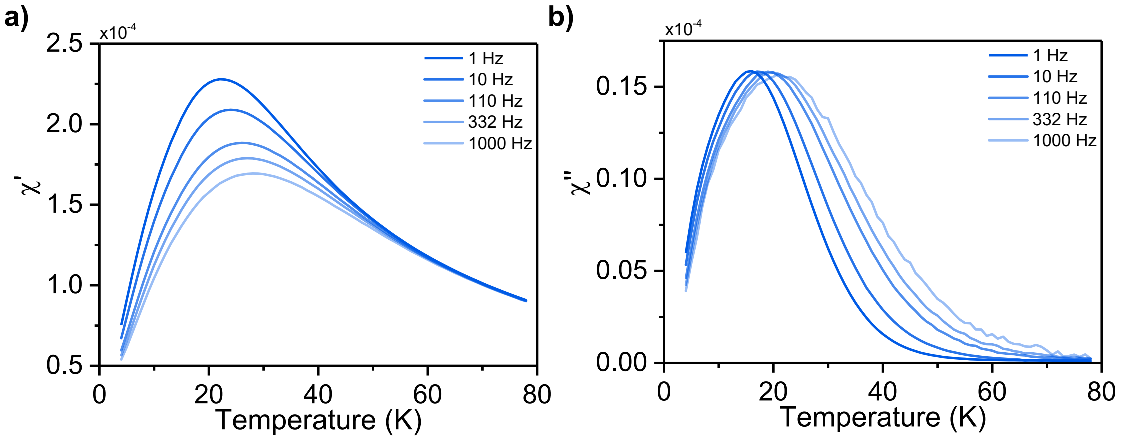

Figure S19. a) In-phase and **(b)** out-of-phase susceptibility at different frequencies of Fe_3O_4NP/CNF.

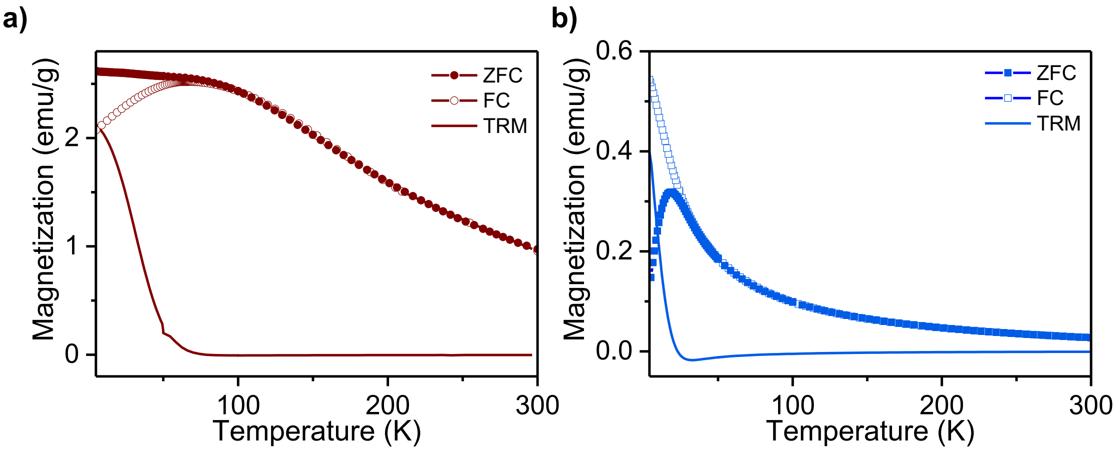

Figure S20. Zero-field-cooled (ZFC)–field cooled (ZF) and thermoremanent (TRM) magnetization curves of **(a)** Fe_3O_4NP and **(b)** Fe_3O_4/CNF.

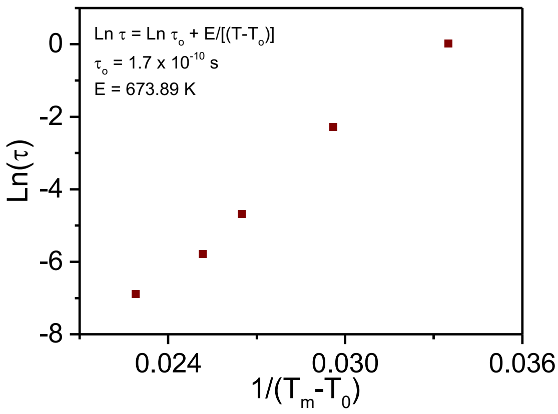

Figure S21. Dependence of relaxation time on temperature for the in-phase χ′ signal for Fe_3O_4NP. Dashed line represents the best fit for the plot of the logarithm of the inverse of the frequency (τ) as a function of the inverse of the peak temperature (T) indicates an interactive system (i.e., dipolar interactions between NP) based on the Vogel-Fulcher law. Here, τ is the relaxation time, τ_0 is a characteristic time constant (related to the attempt frequency by the relation τ_0 = $1/2\pi f_0$), T is the blocking temperature and E the activation energy. Hence, the slope provides an estimate of the activation energy (E) and the y-axis intercept provides an estimate of the τ_o based.

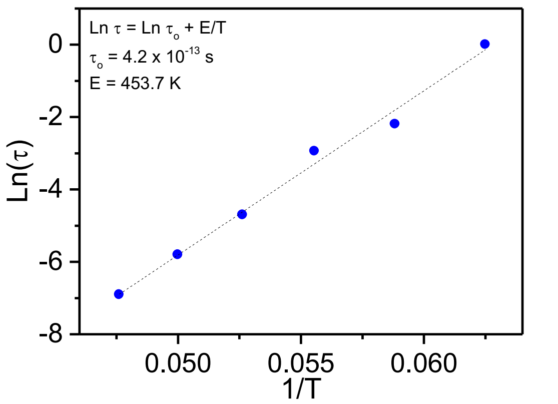

Figure S22. Dependence of relaxation time on temperature for the in-phase χ′ signal for Fe_3O_4/CNF. Dashed line represents the best fit for the plot of the logarithm of the inverse of the frequency (τ) as a function of the inverse of the peak temperature (T) indicates a noninteracting system based on the Arrhenius law. Here, τ is the relaxation time, τ_0 is a characteristic time constant (related to the attempt frequency by the relation τ_0 = $1/2\pi f_0$), T is the blocking temperature and E the activation energy. Hence, the slope provides an estimate of the activation energy (E) and the y-axis intercept provides an estimate of the τ_o based.

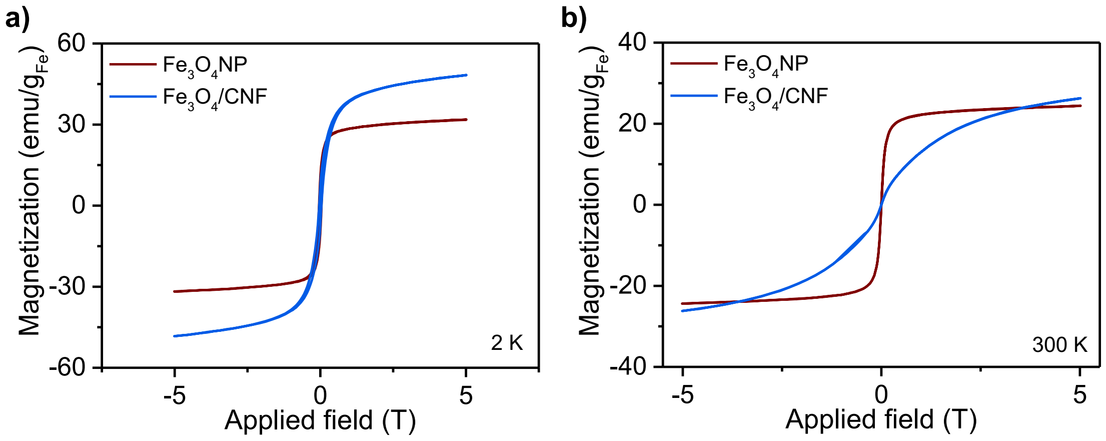

Figure S23. (a) Saturation magnetization of Fe_3O_4NP and Fe_3O_4/CNF at 2 K. **(b)** Saturation magnetization of Fe_3O_4NP and Fe_3O_4/CNF at 300 K. Magnetization is normalized to the total mass of Fe.

9. References

[1] S. Sun, H. Zeng, *J. Am. Chem. Soc.* **2002**, *124*, 8204–8205.

[2] G. A. El-Mahdy, A. M. Atta, H. A. Al-Lohedan, *Molecules* **2014**, *19*, 1713–1731.

[3] Y. S. Li, J. S. Church, A. L. Woodhead, *J. Magn. Magn. Mater.* **2012**, *324*, 1543–1550.

[4] O. N. Shebanova, P. Lazor, *J. Solid State Chem.* **2003**, *174*, 424–430.

[5] N. Sinan, E. Unur, *Mater. Chem. Phys.* **2016**, *183*, 571–579.

[6] P. Arévalo, J. Isasi, A. C. Caballero, J. F. Marco, F. Martín-Hernández, *Ceram. Int.* **2017**, *43*, 10333–10340.

[7] M. Aygün, T. W. Chamberlain, M. del C. Gimenez-Lopez, A. N. Khlobystov, *Adv. Funct. Mater.* **2018**, *28*, 1–15.

[8] A. G. Bannov, N. F. Uvarov, A. V. Ukhina, I. S. Chukanov, K. D. Dyukova, G. G. Kuvshinov, *Carbon N. Y.* **2012**, *50*, 1090–1098.

[9] H. J. Schulte, B. Graf, W. Xia, M. Muhler, *ChemCatChem* **2012**, *4*, 350–355.

[10] E. R. Edwards, E. F. Antunes, E. C. Botelho, M. R. Baldan, E. J. Corat, *Appl. Surf. Sci.* **2011**, *258*, 641–648.

[11] J. S. Ye, X. Liu, H. F. Cui, W. De Zhang, F. S. Sheu, T. M. Lim, *Electrochem. commun.* **2005**, *7*, 249–255.

[12] W. Gruner, *Mikrochim. Acta* **1986**, *88*, 301–309.

[13] A. V. Syugaev, S. F. Lomaeva, A. N. Maratkanova, D. V. Surnin, S. M. Reshetnikov, *Prot. Met. Phys. Chem. Surfaces* **2009**, *45*, 81–88.

[14] I. Castellanos-Rubio, I. Rodrigo, A. Olazagoitia-Garmendia, O. Arriortua, I. Gil De Muro, J. S. Garitaonandia, J. R. Bilbao, M. L. Fdez-Gubieda, F. Plazaola, I. Orue, A. Castellanos-Rubio, M. Insausti, *ACS Appl. Mater. Interfaces* **2020**, *12*, 27917–27929.
